# The Effect of Stress Distribution by Fiberglass Post System With Different Designs on Endodontically Treated Maxillary Central Incisors: A Three-Dimensional Finite Element Analysis

**DOI:** 10.7759/cureus.81545

**Published:** 2025-03-31

**Authors:** Jamshid Usman M, Rohit Raghavan, Hareesh M T, Shajahan P A, Raihana A Latheef S V

**Affiliations:** 1 Prosthodontics, Government Dental College, Kozhikode, IND; 2 Prosthodontics and Crown and Bridge, Royal Dental College, Palakkad, IND; 3 Prosthodontics, Royal Dental College, Palakkad, IND; 4 Dentistry, Explore Medicare, Malappuram, IND

**Keywords:** displacement, fiber post, finite element analysis, parallel post, tapered post, two-stage cylindrical post

## Abstract

Background: The stress distribution pattern in endodontic posts under masticatory load is crucial for optimizing prosthesis design. Limited research exists on the effect of various force vectors on teeth restored with fiber posts of different designs. This study analyzes and compares the stress distribution in endodontically treated maxillary central incisors restored with fiber posts of varying designs using finite element analysis (FEA).

Methods: Three identical 3D finite element models of a maxillary central incisor were created from computed tomography (CT) scan images. Three models with different glass fiber post designs (parallel, tapered, and two-stage cylindrical) were also developed. Stress analysis was conducted using the ANSYS 12.1 software (ANSYS, Inc., Canonsburg, PA, USA) under a physiological load of 70 N, applied at 90°, 45°, and 180° angles to the maxillary incisor restored with a post, core, and crown until the prosthetic complex fractured.

Results: Under vertical loading, the tapered post showed the highest stress concentration at the dentin and cementum levels, with values reaching 5.078 MPa and 3.227 MPa, respectively. Horizontal loading resulted in lower von Mises stress levels at the cancellous bone (4.316 MPa) and within the post (8.385 MPa) for the tapered post. The parallel post demonstrated the least stress in dentin (5.1598 MPa), cementum (10.0258 MPa), and periodontal ligament (PDL) (0.0107 MPa) under oblique loading.

Conclusions: This study suggests that post selection should be based on each clinical scenario's material properties and design considerations. FEA results showed significantly lower stress values in various regions of the tooth-restoration complex when a parallel fiber post was used in endodontically treated maxillary central incisors.

## Introduction

Post-endodontic teeth usually become weak due to structural damage that occurs through dental decay, previous restorations, and mechanical requirements of root canal procedures. The dental profession commonly uses post-and-core restorations to improve both the strength and maintenance longevity of these structures [1]. The use of metal posts has included cast nickel-chromium, prefabricated stainless steel, and titanium materials due to their strong mechanical characteristics and durability. Metallic posts demonstrate excellent effectiveness in clinical use yet present substantial drawbacks because of their high elastic modulus, which generates strain concentrations that increase the danger of root fractures [2]. This drawback has led to a shift in preference toward fiber-reinforced post systems, particularly glass fiber posts, which offer biomechanical advantages, improved esthetics, and a modulus of elasticity closer to that of dentin [3].

Two fundamental groups of prefabricated post systems exist: metallic and non-metallic. Zinc phosphate cement traditionally serves to lute metallic post materials made of stainless steel and titanium alloy, but the root canal system receives adhesive bonding of zirconia, carbon fiber, and glass fiber-reinforced resin composites [4]. The material sciences industry has developed new post systems incorporating carbon, quartz, and glass fiber posts to distribute stress more effectively and prevent post-failure. The integration capability of glass fiber posts with composite resin cores has made them popular, as it results in better load distribution and reduced risk of root fracture [5]. The elastic behavior of fiber posts similar to dentin makes them effective in force distribution and spreads tension between the restoration and post-dentin boundary, thus extending the restoration's lifespan to eight years [6].

Multiple factors make it difficult to evaluate stress distribution in post-restored teeth due to the biomechanical behavior of the restoration, which depends on various elements. Multiple variables determine post-core restoration performance, including post-core materials together with cement type and the presence of periodontal ligament (PDL), alveolar bone characteristics, and occlusal force patterns [7]. The assessment of stress transfer through various post structures makes use of tensile testing along with shear testing and photoelastic analysis as experimental methods [8]. Two-dimensional (2D) stress analysis methodologies that encompass 2D finite element modeling systems fail to replicate both complex tooth structures and detailed occlusal loading dynamics [9]. The implementation of three-dimensional (3D) finite element analysis (FEA) represents a precise method to assess post-and-core restorations' stress patterns. The simulation accuracy of mechanical stresses becomes improved through 3D FEA models as they avoid the structural simplifications of 2D models while delivering better clinical choices to dentists [10].

The post's design determines how stress distributes itself throughout the tooth-restoration complex. The biomechanical response of post configurations to occlusal loading differs based on parallel, tapered, and two-stage cylindrical design types [11]. The distribution of force along root canal walls becomes more uniform with parallel posts because they reduce stress concentrations. Tapered posts duplicate root canal anatomy. However, their apical area tends to accumulate higher stress, which increases the fracture risk of the root. A two-stage cylindrical post design incorporates features from parallel and tapered systems that distribute forces effectively across critical areas [12,13]. The selection of an appropriate post design remains vital. It determines both restoration durability and mechanical stability.

Research using FEA has become a common approach to analyzing post-restored teeth for identifying their stress distribution patterns. Multiple research studies demonstrate that properties from post geometry, materials, and bonding methods determine how stress spreads through dentinal tissue and nearby structures [14].

The study aims to utilize 3D FEA to examine the stress distribution patterns of different fiberglass post designs in endodontically treated maxillary central incisors. The objective is to analyze the stress distribution patterns of three fiberglass post types, including parallel, tapered, and two-stage cylindrical configurations.

## Materials and methods

Study design

Three-dimensional FEA served as the methodology for evaluating endodontically treated maxillary central incisors restored with different fiberglass post designs. The study investigated stress distribution together with concentration patterns in three post types: parallel, tapered, and two-stage cylindrical posts. The stress response was tested with simulated physiological masticatory forces through specific statistical analyses, which determined the post design with the best biomechanical performance regarding stress distribution and both load absorption and fracture resistance for achieving long-term restorative success.

Geometric model construction

A medical imaging system known as computed tomography (CT) generated precise 3D models of maxillary central incisors through sequential image scanning for precise anatomical features. The Materialise's Interactive Medical Image Control System (MIMICS v8.11, Materialise NV, Leuven, Belgium) processed the images to divide different anatomical structures before creating an exact stereolithography (STL) format surface model. SolidWorks software (Dassault Systèmes, Vélizy-Villacoublay, France) allowed the refinement of the model through geometric adjustments, surface optimization, and anatomical details enhancement before meshing.

**Figure 1:**
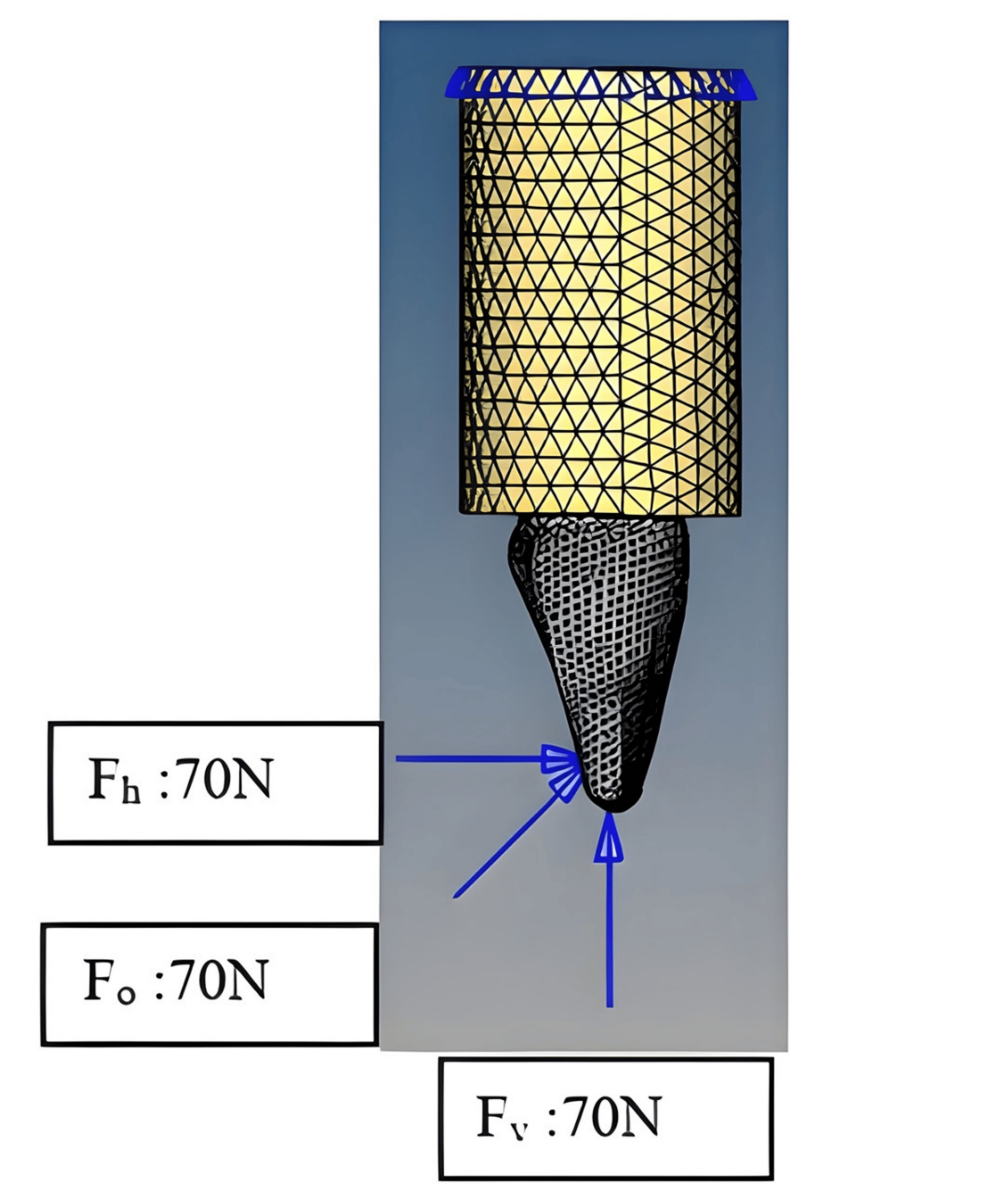
Representation of the constructed geometric model Image Credits: Jamshid Usman

The validity of the built 3D model was confirmed through direct comparisons with published FEA studies and reference standards from standard anatomy. The model required adjustments for the precise replication of physical dimensions found in maxillary central incisor teeth. The completed 3D model contained combinations of enamel, dentin, and cementum along with root canal tissue together with PDL, cortical bone, cancellous bone, and final restoration materials, which included fiberglass posts with luting cement, a composite core, and a ceramic crown. The constructed model is illustrated in Figure 1.

Finite element model generation

Through HyperMesh version 11.0 from Altair HyperWorks (version 11.0) (Troy, MI, USA), the 3D geometric models achieve high-quality finite element meshing that includes optimized nodal connectivity. The design included all necessary dentin and enamel components, PDL, cortical, cancellous bone, fiber post, luting cement and composite core, and an all-ceramic crown. Analysis of stress distribution required a perfect bond between the post and root dentin because it ensured uniform stress distribution.

Material properties

Table 1 presents the material properties used in the study.

**Table 1:** Material properties used in the study

Material	Elastic Modulus (GPa)	Poisson's Ratio	Yield Strength (MPa)	Density (g/cm³)	Fracture Toughness (MPa√m)	Reference
Glass Fiber Post	30.0	0.2	250	2.2	2.0	[1]
Dentin	18.6	0.3	150	1.85	1.5	[2]
Cementum	20.0	0.25	160	1.90	1.2	[3]
Core Material	5.0	0.4	200	1.85	1.8	[4]
Crown Material	12.0	0.3	350	2.0	2.5	[5]
Periodontal Ligament	0.01	0.49	-	1.0	-	[6]
Cortical Bone	17.0	0.35	200	1.8	1.0	[7]

Boundary conditions

The simulation mimicked the physiological conditions of the maxillary central incisor by restraining the alveolar bone in all directions, thus blocking any possible displacement. Adhesive cementation techniques received clinical representation by establishing perfect bonding at the post-dentin interface, which enabled uniform stress transfer. The modeling of the PDL included appropriate elastic properties that represented its shock absorption function. All contacts between the different post-core-cement layers received ideal continuous settings to enable accurate stress distribution analysis.

Load application and stress analysis

The simulation applied a 70 N force at positions and directions that matched natural biting forces, including vertical forces on the incisal edge (90°), oblique forces at 45° 2 mm lingually, and horizontal forces at the same oblique point (180°). The software ANSYS 12.1 (ANSYS, Inc., Canonsburg, PA, USA) examined stress distribution to determine maximum stress concentrations, where von Mises stress values helped identify areas of the highest biomechanical and structural stability.

Meshing and model verification

The simulation models received spatial discretization into tetrahedral high-quality elements, which optimized element number density to provide enhanced numerical performance and numerical precision. A mesh convergence test demonstrated the achievement of mesh refinement until results stabilized, which confirmed a consistent independent stress distribution pattern.

A mesh convergence test was performed to guarantee the accuracy and reliability of the FEA. The Altair HyperMesh 11.0 software was used to mesh the 3D models, and spatial discretization was done using tetrahedral elements. The mesh refinement was performed iteratively until a stable stress distribution pattern was obtained. The convergence criterion was set as a difference of less than 2% in von Mises stress values between successive mesh refinements. After mesh refinement, the results stabilized, meaning that further densification of the mesh did not change the outcome significantly. Therefore, the mesh density used in the final models was validated. The FEA needed this process to maintain high precision and reliability, which reduced computational errors to produce valid biomechanical stress evaluations. The finite element mesh details for different post designs are presented in Table 2.

**Table 2:** Finite element mesh details for different post designs

Post Design	No. of Nodes	No. of Elements
Parallel Post	10,894	52,295
Tapered Post	10,011	51,973
Two-Stage Cylindrical	9,772	50,505

Studied structures and outcome variables

The study evaluated the biomechanical behavior of endodontically treated maxillary central incisors restored with different fiber post designs. The anatomical structures analyzed were dentin, cementum, PDL, cortical bone, cancellous bone, glass fiber post, core material, and crown.

The key outcome variables assessed using FEA were (1) von Mises stress distribution (MPa) to determine the mechanical stress patterns within and between different anatomical and restorative components and (2) displacement (mm) to measure the deformation response of each structure under applied loading conditions in various directions (vertical, oblique, and horizontal). The von Mises stress values were selected as the primary mechanical indicator due to their relevance in evaluating complex stress states and predicting the onset of material failure. The analysis also considered displacement in the X, Y, and Z axes to determine how different post designs influenced structural stability.

Displacement measurement

The displacement values in this study are the total resultant linear deformation (millimeters) of the tooth-restoration system under loading. The vector sums of individual displacements in the X (mesiodistal), Y (apicocoronal), and Z (labiolingual) axes were computed using the ANSYS 12.1 software. It provides a complete understanding of the effect of each post design on the stability and movement of the prosthetic complex under vertical, horizontal, and oblique forces.

Statistical analysis

No inferential statistical tests were performed in this study. The results are based on numerical outputs generated by FEA simulations using ANSYS 12.1. All values reported are descriptive and represent simulated stress and displacement magnitudes under various loading conditions.

Ethical considerations

The study exclusively utilizes computational simulations through FEA software, as it lacks any need for human participants or animal subjects. The research did not require any ethical approval due to its nature. The methodology of this study followed in silico biomechanical research ethics standards to maintain scientific integrity combined with data accuracy standards. The analysis used verified literature materials and boundary conditions to achieve reliable results without ethical problems related to biological experimentation.

## Results

Stress pattern in tapered post

Stress Pattern in Tapered Post Under Vertical Loading

**Figure 2:**
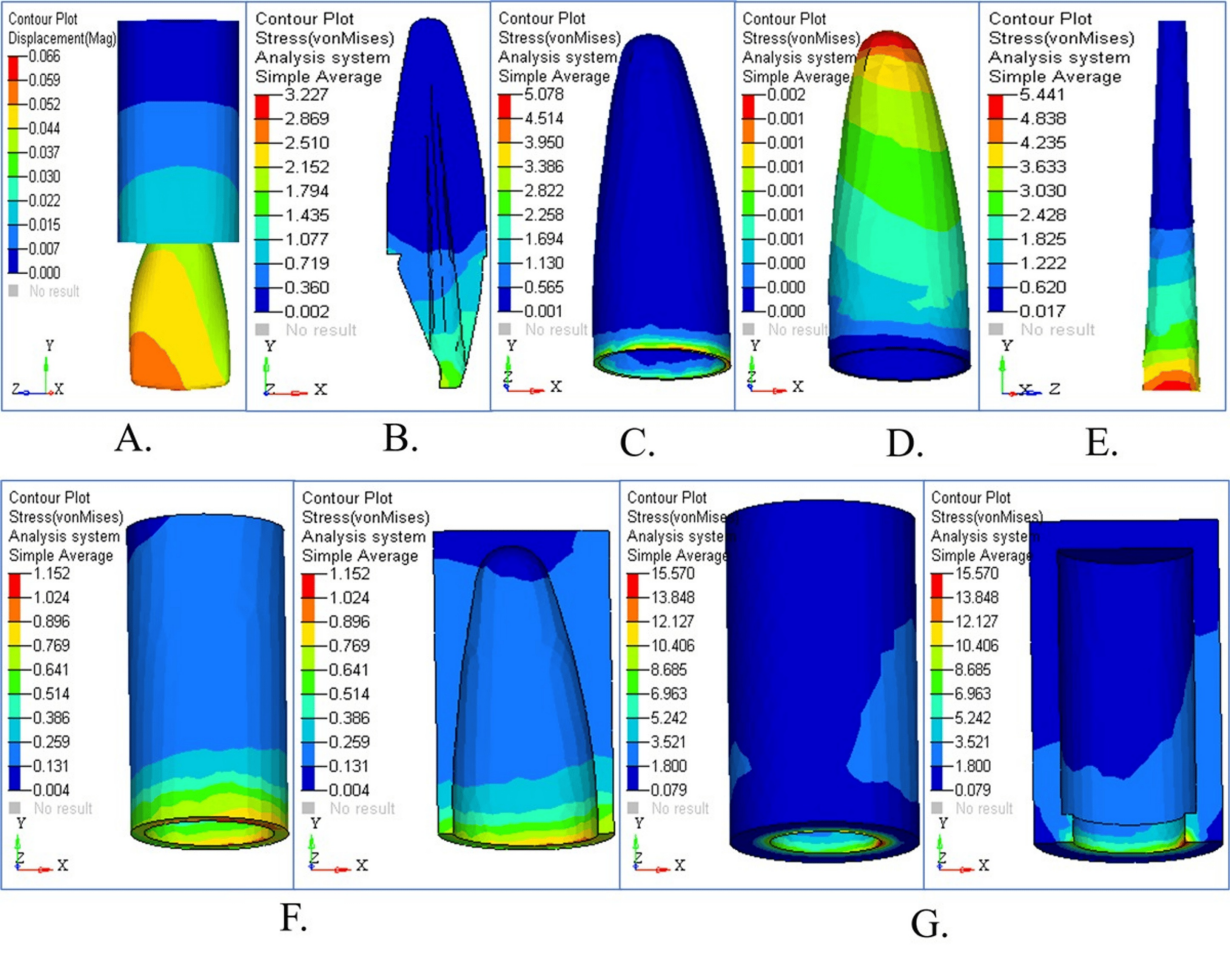
Stress analysis and structural response of tapered post under vertical loading (A) Displacement contour showing the highest deformation in the coronal region; (B) stress distribution in dentin with peak values near the cervical area; (C) cementum stress showing concentration at the interface; (D) periodontal ligament stress with moderate levels; (E) cancellous bone showing balanced stress; (F) cortical bone with maximum stress in the coronal third; (G) combined structural stress overlay for comparative analysis

The stress patterns from vertical loading appear in different areas of the tapered post design, as illustrated in Figure 2. An overview of displacements revealed in Figure 2A demonstrates that the highest values occur in the coronal area. Figure 2B shows the stress distribution in dentin, revealing significant stress concentration in the cervical region. The stress accumulation in cementum, particularly in the interface region, is depicted in Figure 2C. Figure 2D highlights the stress pattern in the PDL, with moderate stress buildup. The stress distribution pattern in cancellous bone stayed balanced compared to the other structures of dentin and cementum (Figure 2E). The coronal third of the root exhibited the highest stress value in the cortical bone (Figure 2F), and Figure 2G overlays structural components for comparison to show their critical stress areas. The maximum stress concentration in the tapered post was found in the apical region of the dentin and cementum under vertical loading (90°). The highest values of von Mises stress were 5.078 MPa in dentin and 3.227 MPa in cementum. Along the root surface, there was also a high level of stress, which indicated that vertical occlusal forces were not well dissipated by the tapered design. The use of a tapered post seems to increase the risk of structural failure or micro-cracks under axial loading.

Stress Pattern in Tapered Post Under Horizontal Loading

**Figure 3:**
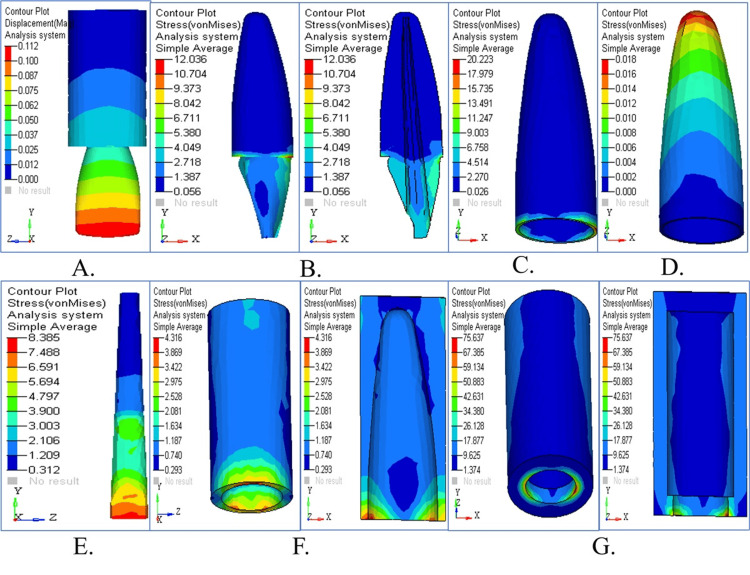
Stress analysis and displacement contour in tapered post under horizontal loading (A) Displacement contour showing significant coronal movement; (B) stress distribution in dentin with high cervical stress; (C) cementum stress concentrated around the cervical area; (D) PDL shows moderate stress levels; (E) cancellous bone with lower stress values; (F) cortical bone experiencing high stress; (G) overall stress map indicating maximum strain regions

The high stress levels at the cervical dentin and cementum under horizontal loading conditions raised the possibility of structural failure (Figures 3B, 3C). Figure 3A illustrates substantial coronal displacement. Figure 3D demonstrates moderate PDL stress levels, but Figures 3E and 3F present the maximum stress levels in cortical bone. The stress distribution map in Figure 3G shows that cortical bone takes the maximum load as it experiences critical strain regions across all structural components, although cancellous bone sustains lower stress levels. Horizontal loading (180°) caused a significant shift of stress towards the mid-root and post-body region. The von Mises stress decreased at the bone level (4.316 MPa) and in the post (8.385 MPa), showing better stress distribution in these zones than in the case of vertical loading. Stress at the dentin-cementum interface remained elevated, however, indicating vulnerability to lateral forces, which may happen in clinical situations; finally, the continuous plate may float away from the cavity wall with excessive instrumentation.

Stress Pattern in Tapered Post Under Oblique Loading

**Figure 4:**
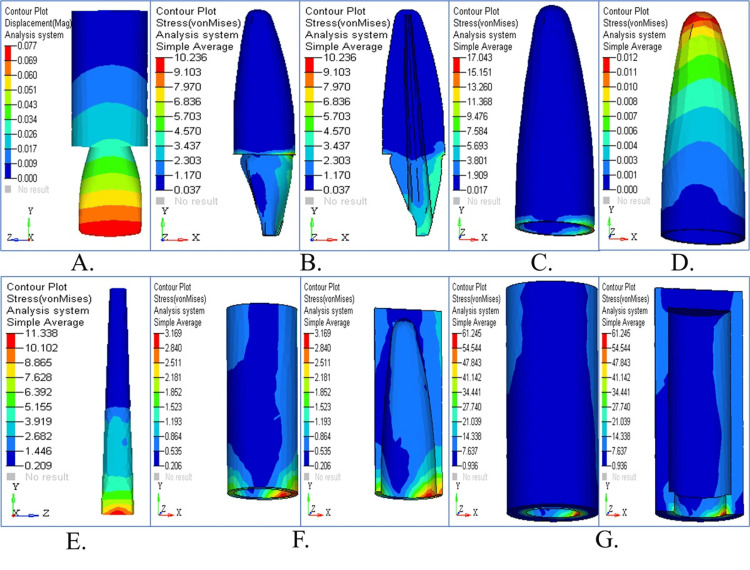
Stress analysis and displacement contour in tapered post under oblique loading (A) Displacement profile under 45° loading; (B) dentin stress under oblique force; (C) cementum stress with notable apical transmission; (D) PDL shows evenly distributed stress; (E) cancellous bone stress minimal; (F) cortical bone shows reduced stress; (G) comprehensive stress overlay showing structural interaction

The maximum stress accumulates in dentin and cementum tissue (Figures 4B, 4C), as these components were susceptible to oblique loading forces. PDL stress shows an even distribution under vertical loading conditions compared to horizontal loading in Figure 4D. The stress level in cancellous bone remains low, thus indicating effective force dispersion in Figure 4E. The stress levels in cortical bone registers are lower than in horizontal loading, which decreases the chance of fractures. The stress distribution areas under oblique forces in detail through the stress map are depicted in Figure 4G. Stress distribution was widespread under oblique forces (45°) across the post, dentin, and PDL. High transmission of stress through the post into surrounding structures was shown as an indication that oblique masticatory loads may amplify root stress in tapered post systems.

Stress pattern in parallel post

Stress Pattern in Parallel Post Under Vertical Loading

**Figure 5:**
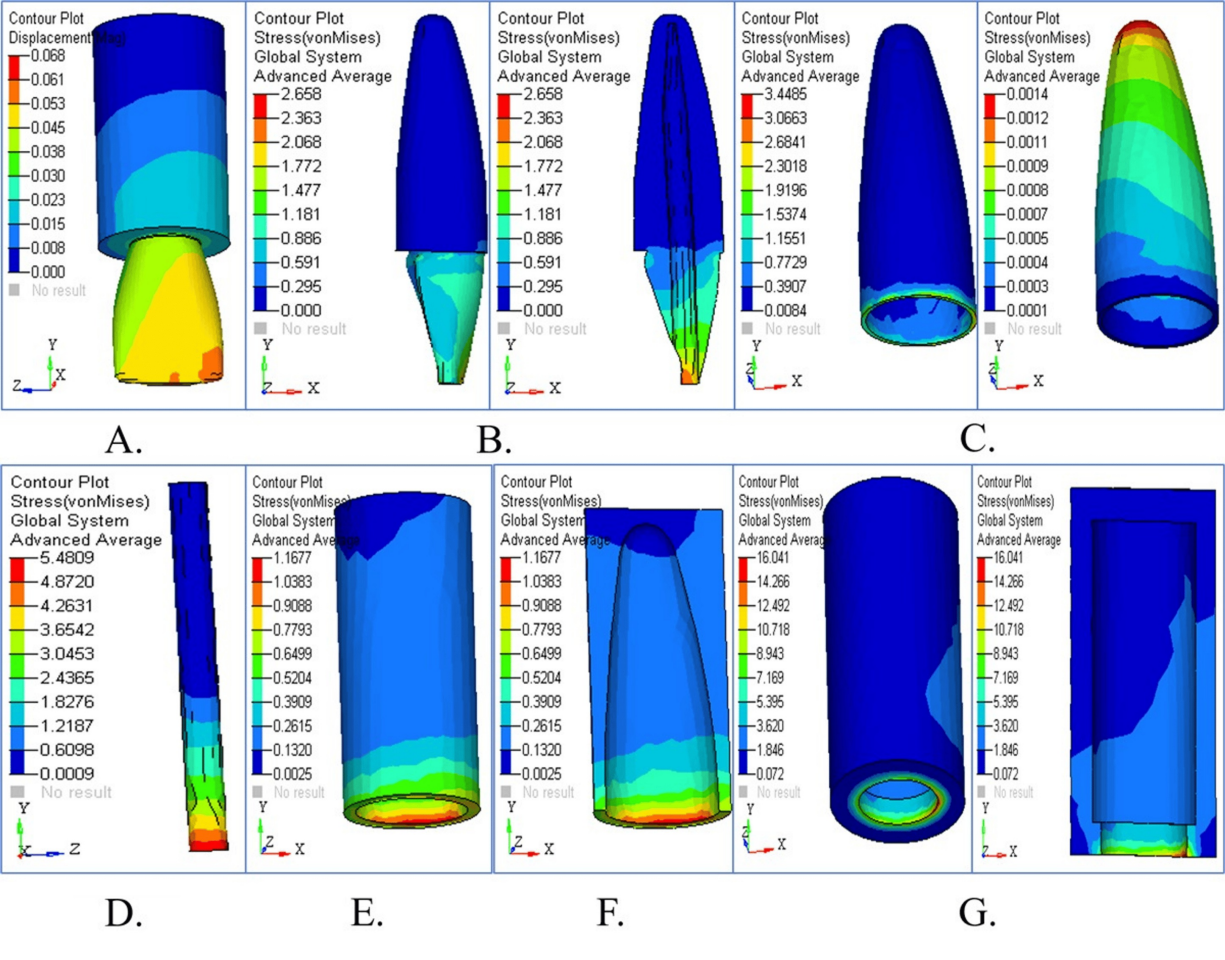
Stress analysis and displacement contour in parallel post under vertical loading (A) Displacement with minimal deformation; (B) stress in dentin distributed uniformly; (C) cementum stress mild across apical third; (D) PDL under low strain; (E) balanced cancellous bone stress; (F) cortical bone under moderate load; (G) overall structure with effective stress control

The stress patterns and displacement movements that occur in parallel posts when they are subjected to vertical force are illustrated in Figure 5. The displacement contour demonstrated small displacement compared to other post types (Figure 5A). The distribution of stress becomes visible (Figures 5B, 5C) as forces are distributed evenly in dentin and cementum. The stress patterns within the PDL are shown in Figure 5D, followed by stress distribution in cancellous and cortical bone (Figures 5E, 5F). The parallel post effectively distributed the forces through its stress patterns, which are combined in Figure 5G. The parallel post showed a better stress distribution under vertical loading (90°) than the tapered one. The coronal dentin and around the post-core interface were at peak stress with lower values than the tapered post scenario. The parallel post shows a reduced stress concentration in the apical third, which means that the axial forces are effectively dissipated and the longevity of the restoration and the surrounding structures is increased.

Stress Pattern in Parallel Post Under Horizontal Loading

**Figure 6:**
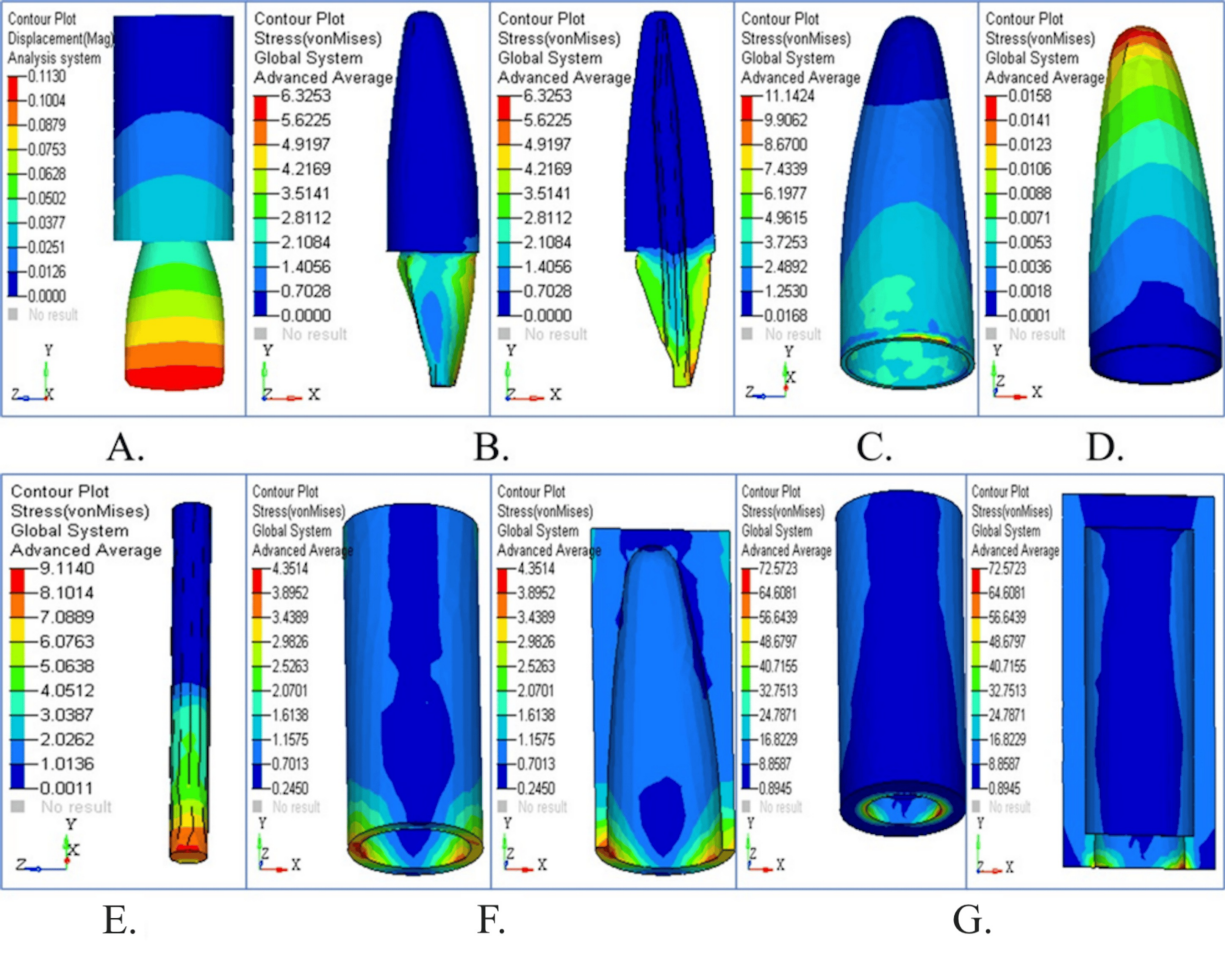
Structural response in parallel post under horizontal loading (A) Moderate coronal displacement; (B) dentin stress localized in the cervical region; (C) cementum stress patterns higher than vertical; (D) PDL showing elevated lateral stress; (E) cancellous bone with even stress distribution; (F) cortical bone under maximum stress; (G) summary map showing optimal force absorption

Figure 6 depicts the stress distribution and displacement patterns that occur when the parallel post model experiences horizontal loading forces. The displacement contour in Figure 6A revealed moderate movement patterns as a result of lateral forces. The cervical location showed maximum stress accumulation in both dentin and cementum (Figures 6B, 6C). The stress levels in PDL shown in Figure 6D rose above those measured with vertical loading. Cortical bone exhibits the maximum stress values (Figures 6E, 6F), while cancellous bone shows its stress patterns. All stress components revealed that parallel posts optimize horizontal force management compared to alternative post types (Figure 6G). The parallel post was subjected to moderate stress levels under horizontal loading (180°), with the stress being localized around the cervical dentin and the PDL. In contrast to the tapered post, stress did not build up significantly in the post body or apical regions. This indicates that the resistance to lateral forces is increased, and the risk of post debonding or root fracture under functional loading is decreased.

Stress Pattern in Parallel Post Under Oblique Loading

**Figure 7:**
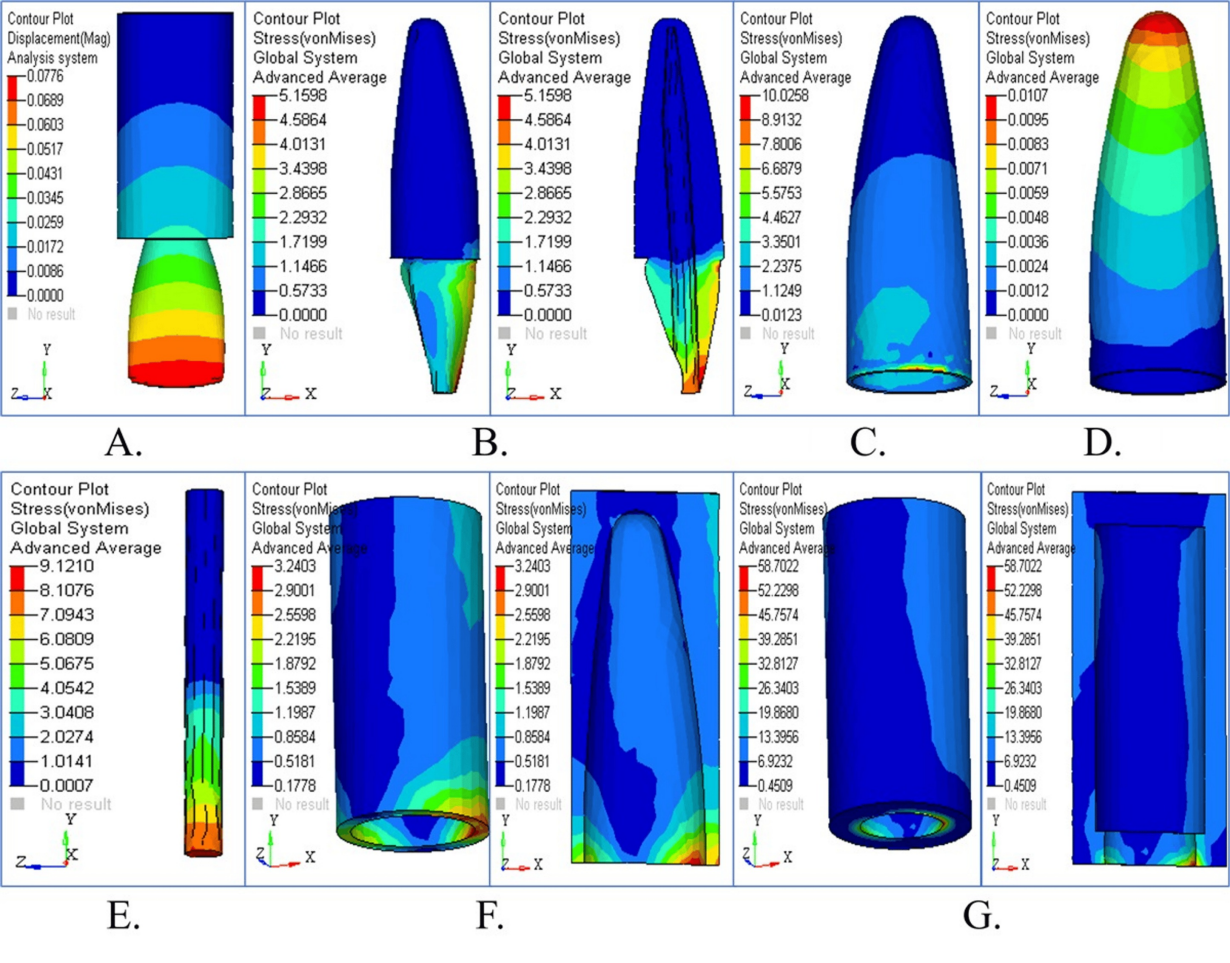
Structural response in parallel post under oblique loading (A) Oblique force leads to moderate displacement; (B) stress in dentin slightly elevated; (C) cementum shows efficient stress dissipation; (D) PDL moderately stressed; (E) low cancellous bone stress; (F) cortical bone with controlled loading; (G) overall overlay highlights structural harmony

Under oblique loading conditions, stress distribution and displacement behavior affect the parallel post model (Figure 7). The post demonstrated noticeable displacement (Figure 7A). The stress concentrations tend to occur in dentin and cementum (Figures 7B, 7C) while demonstrating improved force distribution compared to horizontal loading. PDL stress stayed moderate (Figure 7D), although it surpassed vertical loading levels. The stress analysis revealed that significant stress absorption occurs in cortical bone tissue (Figures 7E, 7F). All stress maps show that the parallel post sustains stable structural integrity during oblique loading conditions (Figure 7G). When compared to other post designs, the lowest von Mises stress was produced by oblique force application (45°) across dentin (5.1598 MPa), cementum (10.0258 MPa), and PDL (0.0107 MPa). In this complex load, the parallel post exhibited optimal biomechanical performance and, therefore, superior stress dissipation at all simulated regions. It was, as such, suitable for clinical situations that incorporate multidirectional occlusal forces.

Stress pattern in cylindrical post

Stress Pattern in Cylindrical Post Under Vertical Loading

**Figure 8:**
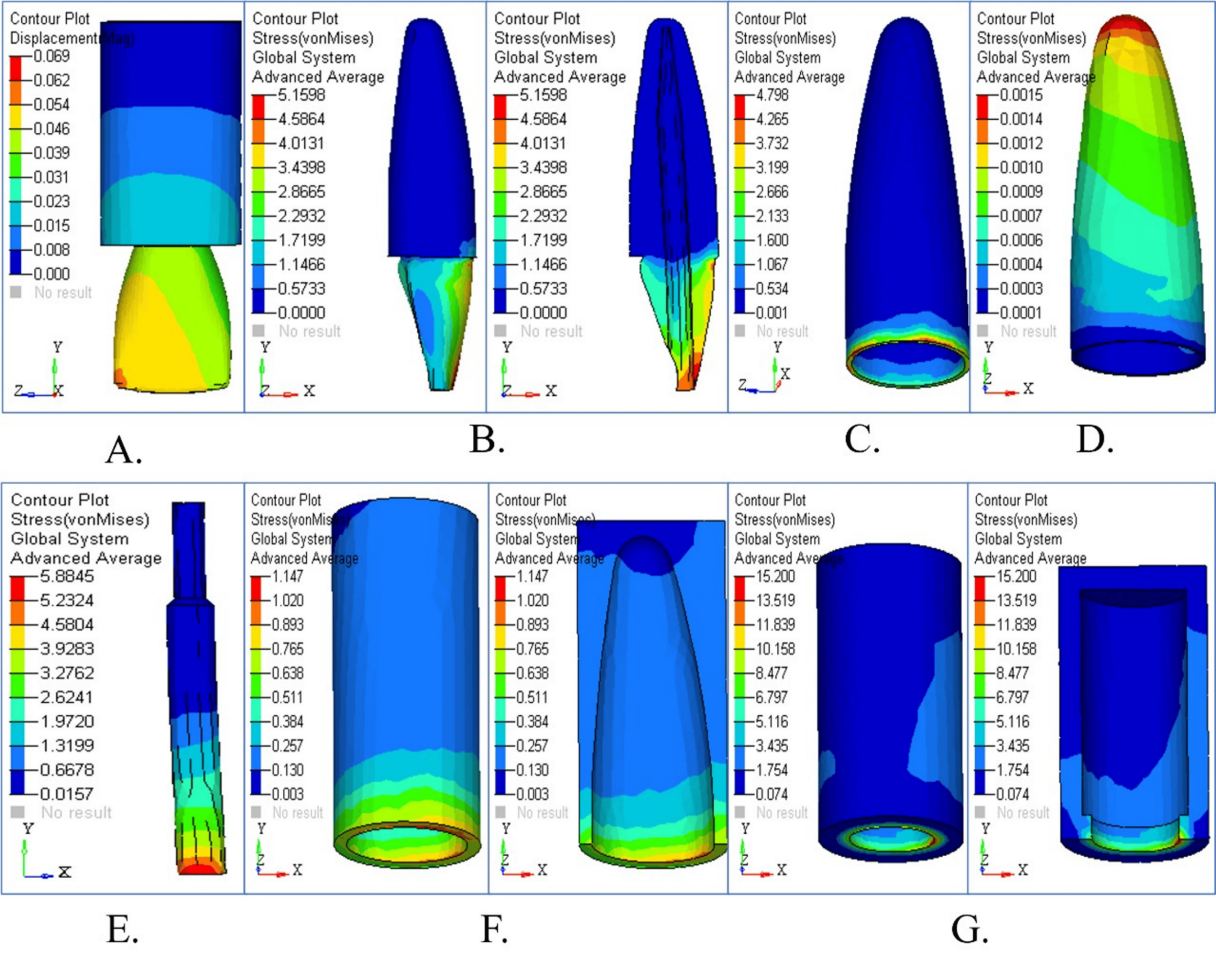
Structural response in cylindrical post under vertical loading (A) Displacement shows minimal post movement; (B) dentin stress spread evenly; (C) cementum displays controlled stress; (D) PDL under low physiological strain; (E) cancellous bone minimally stressed; (F) cortical bone with moderate stress; (G) summary panel showing effective force flow

The stress patterns resulting from the two-stage cylindrical post model, when subjected to vertical loading, are depicted in Figure 8. Figure 8A shows the displacement contour, revealing minimal movement. Dentin and cementum stress accumulation show a more balanced distribution in the observed post designs (Figures 8B, 8C). The stress distribution in the PDL appears in Figure 8D, and cancellous and cortical bone stress is shown in Figures 8E, 8F. The combined stress maps reveal optimal force distribution together with vertical force stability achieved by the two-stage cylindrical post (Figure 8G). The two-stage cylindrical post showed stress concentration under vertical loading (90°) at the junction between the core and the post and moderate stresses toward the apical third of the root. While the stress levels were lower than those of the tapered post, they were greater than those of the parallel post, indicating average performance in distributing axial forces. The mechanical benefit of this design seems to be to redirect stress toward the coronal region.

Stress Pattern in Cylindrical Post Under Horizontal Loading

**Figure 9:**
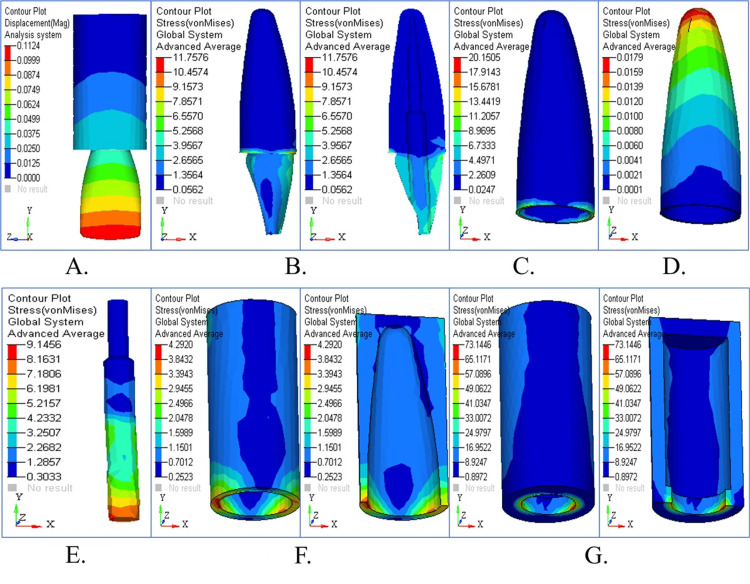
Structural response in cylindrical post under horizontal loading (A) Moderate horizontal displacement observed; (B) dentin stress at cervical level; (C) cementum with concentrated stress zone; (D) PDL showing typical stress contours; (E) cancellous bone shows minimal impact; (F) cortical bone under peak stress; (G) overall panel displays load concentration

Figure 9 illustrates the stress distribution together with displacement patterns of the two-stage cylindrical post subjected to horizontal loading. Figure 9A shows moderate displacement across the model. The maximum stress values appear in the cervical dentin and cementum (Figures 9B, 9C). Stress in PDL tissue (Figure 9D) and the maximum load on cortical bone (Figures 9E, 9F) are noted. All parts of the system achieved an effective stress distribution (Figure 9G). The stress distribution in the cylindrical post system was moderate, with minimal stress in the post and dentin when the post was loaded horizontally (180°). The stress was less concentrated than in the tapered post but more scattered than in the parallel post, especially in the post body and the middle third of the root. It indicates partial effectiveness against lateral forces with a possibility of the occurrence of localized stress peaks that will affect long-term stability.

Stress Pattern in Cylindrical Post Under Oblique Loading

**Figure 10:**
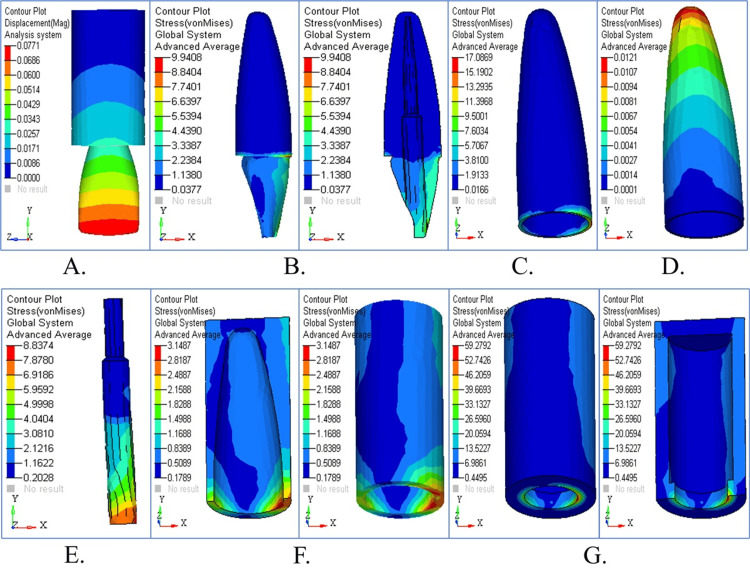
Structural response in cylindrical post under oblique loading (A) Displacement showing intermediate values; (B) dentin stress accumulated cervically; (C) cementum exhibiting localized stress; (D) PDL showing low to moderate stress; (E) balanced cancellous bone stress; (F) cortical bone with stable response; (G) combined structural overlay for post performance

Figure 10 depicts the stress patterns, together with the displacement behavior of the two-stage cylindrical post model when subjected to oblique loading. A medium level of post movement is illustrated in Figure 10A. Figures 10B, 10C highlight stress accumulation in dentin and cementum, particularly in the cervical area. The stress values in the PDL fall within the accepted physiological ranges (Figure 10D). Cancellous bone stress distributions (Figure 10E), together with cortical bone stress distributions in Figure 10F, show cortical bone experiencing less stress than horizontal loading. Figure 10G revealed that the post distributes oblique forces efficiently while sustaining stability by illustrating all stress distributions together. The stress distribution pattern in the two-stage cylindrical post under oblique loading (45°) was intermediate between that of the parallel and tapered posts. A mixed stress behavior was evident at the post-dentin interface and in the cementum. It was better than the tapered post but did not outperform the parallel design in reducing stress in the tooth-restoration complex.

Comparative stress distribution in different fiber post models under various loading conditions

Table 3 presents the stress value comparison of different structural regions between tapered, parallel, and two-stage cylindrical fiber post models when subjected to vertical, horizontal, and oblique loading loads. Under horizontal loading, stress achieved its maximum values throughout all post test models, particularly in cortical bone together with dentin and cementum. Stress values in the parallel post were lower than in other post types, and the two-stage cylindrical post showed stress distribution that remained balanced. The PDL showed minimal stress levels consistently, which demonstrates its role in force absorption.

**Table 3:** Maximum stress distribution across tapered, parallel, and two-stage cylindrical fiber post models (MPa)

Loading Direction	Post Model	Dentin	Cementum	PDL	Post	Cancellous Bone	Cortical Bone
Vertical	Tapered	3.227	5.078	0.002	5.441	1.152	15.570
	Parallel	2.658	3.4485	0.0014	5.4809	1.1677	16.041
	Two-Stage	3.302	4.79	0.0015	5.88	1.15	15.2
Horizontal	Tapered	12.036	20.223	0.018	8.385	4.316	75.637
	Parallel	6.3253	11.1424	0.0158	9.1140	4.3514	72.5723
	Two-Stage	11.76	20.15	0.0179	9.14	4.29	73.14
Oblique	Tapered	10.236	17.043	0.012	11.338	3.169	61.245
	Parallel	5.1598	10.0258	0.0107	9.1210	3.2403	58.7022
	Two-Stage	9.94	17.087	0.0121	8.84	3.15	59.28

Comparative displacement analysis in different fiber post models under various loading conditions

Table 4 presents the maximum displacement evaluation of various fiber post models. Horizontal loading produced the greatest displacement amounts among all post models while demonstrating the difficulties of working against lateral forces. Post stability appeared highest in the two-stage cylindrical model, as it demonstrates minimum displacement variation. The force distribution system of vertical loading produced the most effective results for displacement reduction. The distribution of stress under oblique loading appeared balanced as the measured values fell between the other post model results.

**Table 4:** The von Mises stress and displacement values for different post designs under various loading conditions Note: All values are descriptive results from finite element analysis (FEA) simulations. No statistical tests were conducted.

Post Design	Loading Direction	von Mises Stress (MPa)	Displacement (mm)
Tapered Post	Vertical	Dentin: 3.227, Cementum: 5.078, PDL: 0.002	0.066
	Horizontal	Dentin: 12.036, Cementum: 20.223, PDL: 0.018	0.112
	Oblique	Dentin: 10.236, Cementum: 17.043, PDL: 0.012	0.077
Parallel Post	Vertical	Dentin: 2.658, Cementum: 3.4485, PDL: 0.0014	0.068
	Horizontal	Dentin: 6.3253, Cementum: 11.1424, PDL: 0.0158	0.113
	Oblique	Dentin: 5.1598, Cementum: 10.0258, PDL: 0.0107	0.077
Two-Stage Cylindrical	Vertical	Dentin: 3.302, Cementum: 4.79, PDL: 0.0015	0.069
	Horizontal	Dentin: 11.76, Cementum: 20.15, PDL: 0.0179	0.112
	Oblique	Dentin: 9.94, Cementum: 17.087, PDL: 0.0121	0.077

Stress distribution in different fiber posts under vertical loading

**Figure 11:**
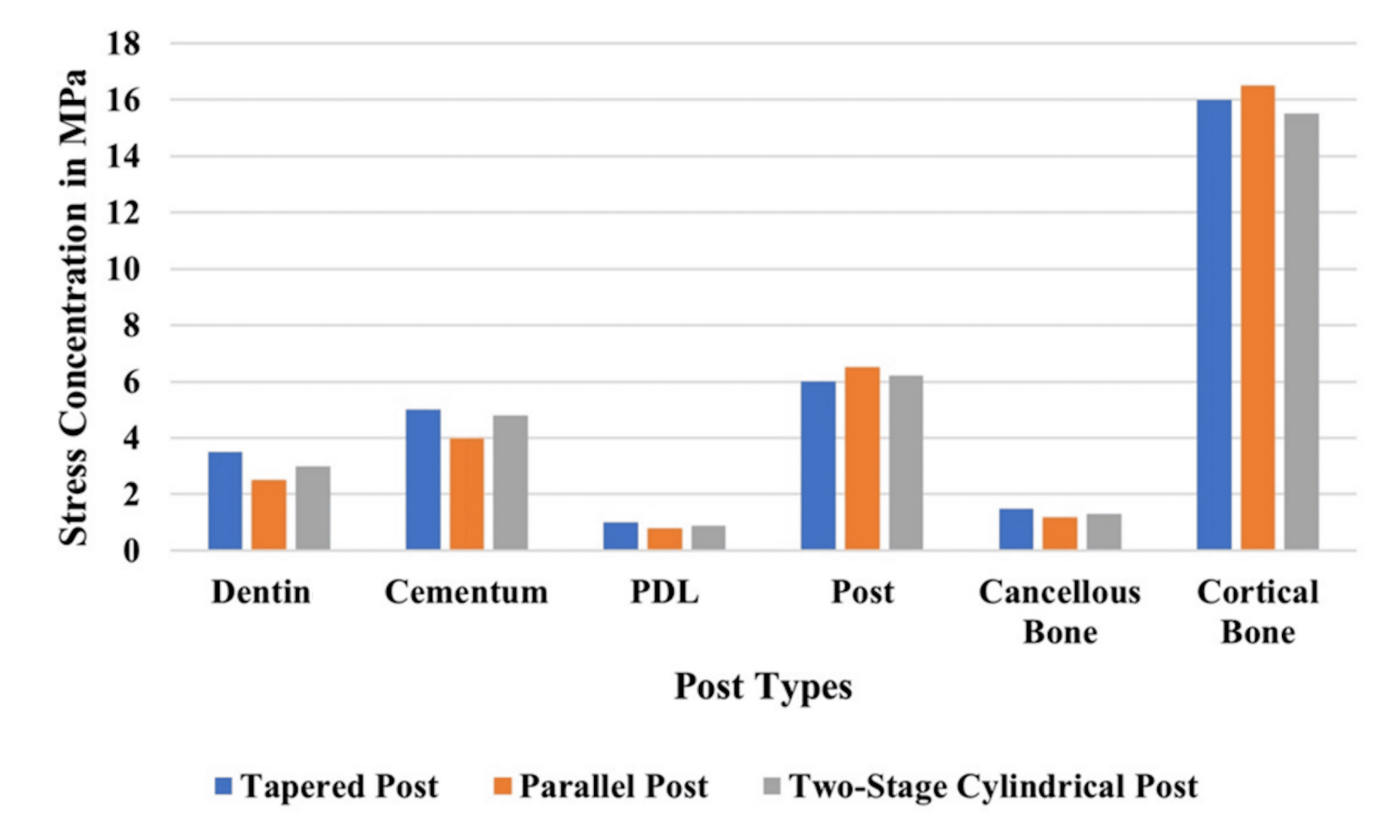
Comparison of stress distribution under vertical loading

Different parts of the structure reveal their stress distribution patterns under horizontal loading (Figure 11). The tapered post design generated the greatest amount of stress in dental structures as well as cortical bone, yet the parallel post demonstrated the lowest stress levels in every structure. The parallel post exhibited the lowest stress levels in cementum, while the PDL and cancellous bone stress levels remained equivalent between all post designs.

Stress distribution in different fiber posts under horizontal loading

**Figure 12:**
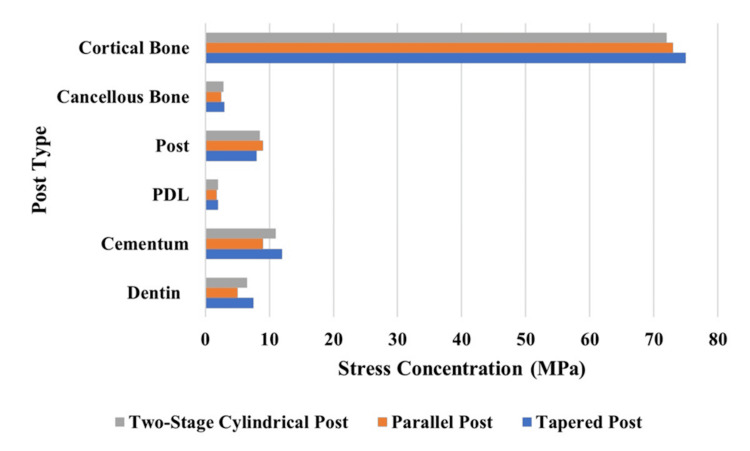
Stress concentration (MPa) under horizontal loading

Figure 12 illustrates how various parts of a structure respond to vertical loads with stress distribution patterns. The tapered and two-stage cylindrical posts generated the most dentinal stress, but the parallel post produced minimum cemental stress. The stress distribution in PDL remained equivalent among every post design. The parallel post design resulted in maximum stress for cortical bone, while cancellous bone stress remained equal across all structural models.

Stress distribution in different fiber posts under oblique loading

**Figure 13:**
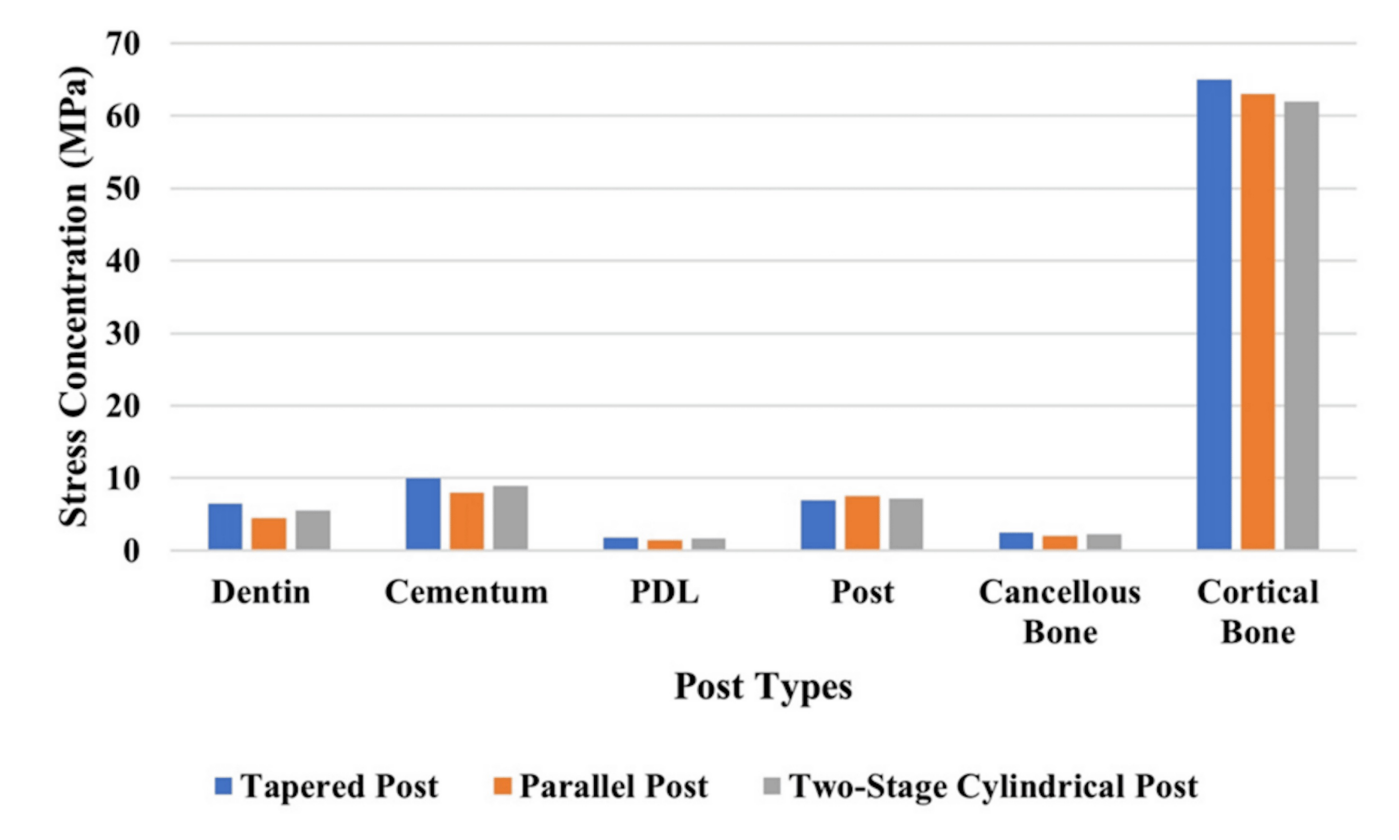
Stress concentration (MPa) under oblique loading

Figure 13 demonstrates the structural elements' stress when oblique loading forces are experienced. Under oblique loading conditions, the tapered post design applied the most stress to dentin and cortical bone, while the parallel post applied the least stress to both parts. The stress on cementum reached its minimum level when using parallel posts, but PDL and cancellous bone stress showed similar patterns between all post models.

## Discussion

The study assessed fiberglass post designs in maxillary central incisors through 3D FEA analysis to determine how these designs distribute stress in endodontically treated teeth. In the study, the stress distribution in endodontically treated maxillary central incisors was restored with three different fiber post designs (parallel, tapered, and two-stage cylindrical) under vertical (90°), oblique (45°), and horizontal (180°) loading conditions that were compared in depth. Three-dimensional FEA of post-endodontic restorations results in the finding that post conditioning has a large effect on stress propagation, mechanical stability, and long-term prognosis of post-endodontic restorations. The analysis is then used to highlight that parallel post system, tapered, and two-stage cylindrical posts distribute forces more unevenly, which results in stress accumulation at the dentin-cementum junction and lower stress redistribution compared to a tapered post or two-stage cylindrical post. Figures 2-13 and Tables 2-3 further support these biomechanical insights.

Stress analysis of various post designs shows parallel posts giving the most favorable stress distribution, particularly under vertical loading (90°). The lowest von Mises stress in dentin and cementum was with the parallel post (2.658 MPa and 3.4485 MPa, respectively) and showed optimal force transmission and reduced fracture risk. The tapered post experienced greater stress accumulation (3.227 MPa in dentin and 5.078 MPa in cementum), which means that microfractures are more likely to occur in the cervical region. Intermediate stress values (3.302 MPa dentin, 4.79 MPa cementum) of the two-stage cylindrical post indicated partial improvement over the tapered post; however, it did not exceed the parallel post. These findings are from previous studies showing that parallel posts have lower stress concentrations because they are capable of spreading occlusal forces more evenly along the root canal walls, thus reducing the risk of mechanical failure [15,16].

All post designs had higher stress concentrations under horizontal loading (180°), corresponding to lateral masticatory forces and bruxism-related stress, especially in cortical bone. The tapered post resulted in the highest stress (75.637 MPa in cortical bone and 20.223 MPa in cementum) and the highest risk of root fracture. The lowest stress levels (72.5723 MPa in cortical bone, 11.1424 MPa in cementum) were shown by the parallel post, verifying its biomechanical superiority to resist lateral force absorption. The stress pattern of the two-stage cylindrical post was similar to the tapered post, except with a slightly reduced peak stress (73.14 MPa in cortical bone, 20.15 MPa in cementum), confirming that it is still susceptible to failures due to horizontal force. These results agree with previous literature, which indicates that tapered posts experience stress peaks at the cervical region and are more susceptible to vertical fracture and post debonding under high lateral forces [17,18].

Stress accumulation in dentin and cementum under oblique loading (45°) was intermediate compared to vertical and horizontal loading. This parallel post showed the least stress (5.1598 MPa in dentin and 10.0258 MPa in cementum) due to its better capability of distributing stress evenly. The stress values in the tapered post were the highest (10.236 MPa in dentin and 17.043 MPa in cementum), and this further supported the fact that the cervical interface of the tapered post is the most susceptible to stress concentration [19,20]. Modest stress values (9.94 MPa in dentin, 17.087 MPa in cementum) were recorded for the two-stage cylindrical post, which were higher than those recorded for the parallel post, indicating that the two-stage cylindrical post showed suboptimal biomechanical performance. These results are consistent with previous studies that post designs that run parallel to the root surface prolong the restoration longevity by reducing structural compromise [21,22].

Insights on restoration stability and retention under occlusal forces are obtained from post displacement behavior. The biomechanical challenge of lateral forces was reinforced by maximum displacement under horizontal loading. Higher displacement (0.112 mm) in tapered posts indicated an increased risk of failure due to post debonding. The parallel post had better retention in comparison to the parallel post, with the lowest displacement (0.068 mm under vertical loading and 0.113 mm under horizontal loading) and was the most stable design for long-term clinical use. Displacement patterns of the two-stage cylindrical post were found to be similar to that of the tapered post, which indicated the moderate performance of the post in resisting post loosening and micro-movements. These findings agree with the findings of previous authors that posts that run parallel to the root canal display greater stability as they can engage a greater amount of dentinal surface and less lateral movement within the root canal [23,24].

In this study, the non-invasive yet precise biomechanical study of stress in post-endodontic restoration was performed using FEA. CT imaging-based geometric models, with high resolution to enable realistic anatomical replication of clinical applicability, were developed. It is noted that the PDL stress values across all post designs were consistently low, indicating its role as a shock absorber, limiting the transmission of excessive force. This observation has previously been verified by biomechanical studies suggesting PDL biomechanics as important instruments in reducing excessive occlusal loadings to maximally restored teeth, which otherwise could cause overload-related stress fractures and mechanical failure [25].

These results clinically show that post selection should not only be based on material properties but also on stress distribution patterns and biomechanical compatibility with the remaining dentin. Parallel posts provide the best mechanical stability and should be the post of choice for restoring endodontically treated maxillary central incisors in load-bearing cases. For high-stress applications such as bruxism patients, tapered posts, although commonly used for anatomical adaptation, have greater displacement and higher stress accumulation than posts with a cylindrical design. These findings imply that stress propagation should be considered in the selection of post designs instead of the traditional assumption of casting tapered posts being more anatomically compatible with the root canals [26,27].

Hence, this study supports that a biomechanically sound approach to post selection is required for endodontic restorations. As fiber-reinforced composite posts are still a material of preference, post geometry cannot be disregarded in terms of mechanical stability. The findings from FEA-based stress distribution studies should be integrated into the decision-making process of clinicians so that the restorations are not only esthetically and structurally sound but also functionally durable. Post selection should be chosen for each patient based on his unique occlusal forces, residual dentin volume, and functional demands to produce the best possible long-term treatment outcome. These findings can be further enhanced by further studies, including clinical trials and in vivo data collection, closer to the applicability to routine dental practice.

Limitations

This study was thus based on a computational simulation utilizing FEA, and while such simulations are highly informative, they also have limitations. They are developed from idealized CT scan images and may inaccurately represent anatomical variations of natural dentition and periodontium structures. An isotropic and homogeneous material assumption was made, but the behavior of biological tissues is generally complex. Moreover, loading conditions were static and did not take into consideration dynamic masticatory forces or fatigue with time. No statistical analysis was conducted, and all results were interpreted descriptively from the FEA output. Additionally, factors such as clinical interface bonding quality, response to biological healing, and occlusal variations of individuals could not be simulated. As such, the findings are of biomechanical interest, but clinical validation through in vivo or in vitro studies is recommended.

Future perspective

The development of future research needs to include individual FEA models for tooth analysis that incorporate patient-specific root characteristics as well as bone density measurements and studies about bite forces for improved realistic clinical evaluations. The durability of different post designs needs to be confirmed through systematic clinical trials that extend over extended periods. New research needs to examine nanofiber-reinforced posts and advanced adhesive systems because they may improve both stress absorption and mechanical stability. AI predictive models, alongside biomechanical simulations and individual patient characteristics, would help select optimal posts according to future dental practice requirements.

## Conclusions

This study shows that fiber post design directly affects both stress distribution and structural integrity of endodontically treated maxillary central incisors. A parallel post design achieved the best biomechanical features by generating minimal stress in dentin (2.658 MPa) and cementum (3.4485 MPa) while showing reduced displacement (0.068 mm) in vertical loading. With its taper design, the post produced the highest stress levels that raised the potential for root fractures, specifically under horizontal loading conditions. Stress absorption and distribution performance of the two-stage cylindrical post was found to be satisfactory, but the recorded stress levels exceeded those of the parallel post. These comparisons are based on numerical trends observed in the simulation outputs and are not derived from statistical hypothesis testing. The research findings show parallel posts offer superior clinical effectiveness for reducing mechanical failure possibilities. The research demonstrates that selecting an ideal post design leads to extended durability of post-endodontic restorations. The recommendations for future research indicate that clinical trials and patient-specific conditions should be studied to validate and improve post placement strategies for better long-term outcomes.

## References

[1] Mannocci F, Bitter K, Sauro S, Ferrari P, Austin R, Bhuva B (2022). Present status and future directions: the restoration of root filled teeth. Int Endod J.

[2] Bhaktikamala A, Chengprapakorn W, Serichetaphongse P (2022). Effect of different post materials and adaptability on fracture resistance and fracture mode in human endodontically treated teeth. Int J Dent.

[3] Alshabib A, Abid Althaqafi K, AlMoharib HS, Mirah M, AlFawaz YF, Algamaiah H (2023). Dental fiber-post systems: an in-depth review of their evolution, current practice and future directions. Bioengineering (Basel).

[4] Heboyan A, Vardanyan A, Karobari MI (2023). Dental luting cements: an updated comprehensive review. Molecules.

[5] Kubo M, Komada W, Otake S, Inagaki T, Omori S, Miura H (2018). The effect of glass fiber posts and ribbons on the fracture strength of teeth with flared root canals restored using composite resin post and cores. J Prosthodont Res.

[6] Zarow M, Vadini M, Chojnacka-Brozek A (2020). Effect of fiber posts on stress distribution of endodontically treated upper premolars: finite element analysis. Nanomaterials (Basel).

[7] Huang M, Wang B, Zhang K, Yan X, Chen Z, Zhang X (2024). Comparative analysis of stress distribution in residual roots with different canal morphologies: evaluating CAD/CAM glass fiber and other post-core materials. BMC Oral Health.

[8] Schürger B, Pástor M, Frankovský P, Lengvarský P (2025). Photoelasticity as a tool for stress analysis of re-entrant auxetic structures. Applied Sciences.

[9] Mehri Sofiani F, Farahani BV, Belinha J (2024). Elasto-static analysis of composite restorations in a molar tooth: a meshless approach. Polymers (Basel).

[10] Kharboutly NA, Allaf M, Kanout S (2023). Three-dimensional finite element study of endodontically treated maxillary central incisors restored using different post and crown materials. Cureus.

[11] Mohan M, Mohammad L, Cholayil N, Vats S, Salman Kuttikkodan M, Kodumbilayiparambil Anto J (2024). Stress distribution on maxillary canines following restoration with different dimensions of metal and fiber posts: a finite element study. Cureus.

[12] Puleio F, Lo Giudice G, Militi A, Bellezza U, Lo Giudice R (2022). Does low-taper root canal shaping decrease the risk of root fracture? A systematic review. Dent J (Basel).

[13] Boțilă MR, Popa DL, Mercuț R, Iacov-Crăițoiu MM, Scrieciu M, Popescu SM, Mercuț V (2024). A finite element method study of stress distribution in dental hard tissues: impact of access cavity design and restoration material. Bioengineering (Basel).

[14] Kochar SP, Reche A, Paul P (2022). The etiology and management of dental implant failure: a review. Cureus.

[15] Iosif L, Dimitriu B, Niţoi DF, Amza O (2023). Endodontic dentistry: analysis of dentinal stress and strain development during shaping of curved root canals. Healthcare (Basel).

[16] Gulabivala K, Ng YL (2023). Factors that affect the outcomes of root canal treatment and retreatment—a reframing of the principles. Int Endod J.

[17] Alamdari Mahd M, Moeiny P, Heshmat H, Askarizadeh N (2023). In vitro comparison of fracture resistance of severely damaged primary anterior teeth restored with different post and core systems. Int J Dent.

[18] de Morais DC, Butler S, Santos MJ (2023). Current insights on fiber posts: a narrative review of laboratory and clinical studies. Dent J (Basel).

[19] Lazari PC, Oliveira RC, Anchieta RB, Almeida EO, Freitas Junior AC, Kina S, Rocha EP (2013). Stress distribution on dentin-cement-post interface varying root canal and glass fiber post diameters. A three-dimensional finite element analysis based on micro-CT data. J Appl Oral Sci.

[20] Madfa AA (2023). Effect of dental glass fiber posts on root stresses and fracture behavior of endodontically treated maxillary central incisors: a finite element analysis study. Cureus.

[21] Baba NZ, White SN, Bogen G (2017). Restoration of endodontically treated teeth. Endodontic Prognosis.

[22] Tallarico M, Ortensi L, Martinolli M (2018). Multicenter retrospective analysis of implant overdentures delivered with different design and attachment systems: results between one and 17 years of follow-up. Dent J (Basel).

[23] Zanza A, Reda R, Testarelli L (2023). Endodontic orthograde retreatments: challenges and solutions. Clin Cosmet Investig Dent.

[24] Peters OA, Arias A (2022). Rotary and reciprocating motions during canal preparation. Eur Endod J.

[25] Duanmu Z, Liu L, Deng Q, Ren Y, Wang M (2021). Development of a biomechanical model for dynamic occlusal stress analysis. Int J Oral Sci.

[26] Vikhe DM (2021). Restoration of endodontically treated teeth. Clin Concepts Pract Manag Dent.

[27] Prakash J, Golgeri MS, Haleem S, Kausher H, Gupta P, Singh P, C SG (2022). A comparative study of success rates of post and core treated anterior and posterior teeth using cast metal posts. Cureus.

